# Q fever outbreak in a slaughterhouse in Brazil: description and decade-later investigation

**DOI:** 10.1007/s15010-026-02922-8

**Published:** 2026-08-24

**Authors:** Danilo A. França, Igor S. Silito, Matheus Pasini-Martins, Leila C. Saad, Tais B. Carvalho, Milenne U. S. Dias, Rodrigo N. Angerami, Fabiana C. P. Santos, Silvia Colombo, Marcelo B. Labruna

**Affiliations:** 1 Department of Preventive Veterinary Medicine and Animal Health, University of São Paulo, São Paulo, Brazil; 2 School of Public Health, University of São Paulo, São Paulo, Brazil; 3 Municipal Health Secretariat, Department of Health Surveillance, Barbosa, São Paulo Brazil; 4 Epidemiological Surveillance Center, Epidemiological Surveillance Group, São Paulo State Health Secretariat, Disease Control Coordination, São Paulo, Brazil; 5 Epidemiological Surveillance Service, University of Campinas, Hospital of Clinics, Campinas, Brazil; 6 Adolfo Lutz Institute, Vector-Borne Diseases Center, São Paulo, Brazil

**Keywords:** *Coxiella burnetii*, Occupational exposure, Chronic infection, Endocarditis

## Abstract

**Purpose:**

To describe the first Q fever outbreak in Brazil and the long-term clinical and serological outcomes of affected individuals ten years after the outbreak.

**Methods:**

A follow-up investigation was conducted among patients involved in a Q fever outbreak that occurred in 2015 in a slaughterhouse in Barbosa, São Paulo State, Brazil. Clinical records from the acute phase were reviewed, and patients were reevaluated ten years later through clinical assessment, review of medical history, serological testing, and molecular analysis.

**Results:**

The outbreak affected 17 of 40 slaughterhouse workers (attack rate = 42.5%) and was likely associated with the inhalation of *Coxiella burnetii* during the slaughter of pregnant cows. Patients presented with prolonged fever, headache, myalgia, pneumonia, hepatitis, and hematuria; six required hospitalization. Ten years later, 14 patients were reevaluated. Of these, 10 developed complications compatible with chronic Q fever, including cardiac and respiratory disorders, as well as persistent fatigue. One woman experienced a premature birth after becoming pregnant following the outbreak. In 2025, one patient presented a phase I antibody endpoint titer of 1:1024 and a history of endocarditis, suggesting persistent infection.

**Conclusion:**

This study provides a unique description of the long-term clinical and serological outcomes of workers involved in the first documented Q fever outbreak in Brazil and emphasizes the importance of post-outbreak surveillance and prolonged clinical follow-up.

**Supplementary Information:**

The online version contains supplementary material available at https://doi.org/10.1007/s15010-026-02922-8.

## Introduction

Q fever is an underestimated zoonosis caused by *Coxiella burnetii* and transmitted mainly through the airborne route, either from infected animals or contaminated environments. Ruminants are currently considered the main sources of infection, especially during parturition or abortion events [1]. Human diagnosis is based on serological testing, with the indirect immunofluorescence assay (IFA) considered the reference method, while doxycycline is the treatment of choice for acute infection [2]. The main preventive measures include the use of personal protective equipment, biosafety practices in occupational settings, and early recognition of suspected cases [3].

Q fever outbreaks have already been reported in several locations [4–12], including outbreaks in slaughterhouses [13–18]. In Brazil, Q fever is highly underestimated and is rarely considered a possible cause of febrile illness by healthcare professionals, a reality observed in most countries [19]. Cases of endocarditis and persistent fever caused by *C. burnetii* have already been described in Brazil, serving as a warning for undiagnosed and untreated cases over time [20, 21].

In this study, we report the first Q fever outbreak in Brazil. To date, no other outbreak has been reported in the country. In addition to describing the outbreak, a decade-later investigation was conducted in 2025 to describe the long-term clinical and serological outcomes of the affected workers.

## Methods

### Ethics statement

The workers were informed that their coded data would be used for the study and for publication. All research participants signed informed consent forms and received their test results, as well the advice to seek medical evaluation regarding the infection follow-up and treatment if necessary. This study was approved by the Human Research Ethics Committee of the University of São Paulo (protocol number 7.843.511).

### Study setting and outbreak investigation

**Fig. 1:**
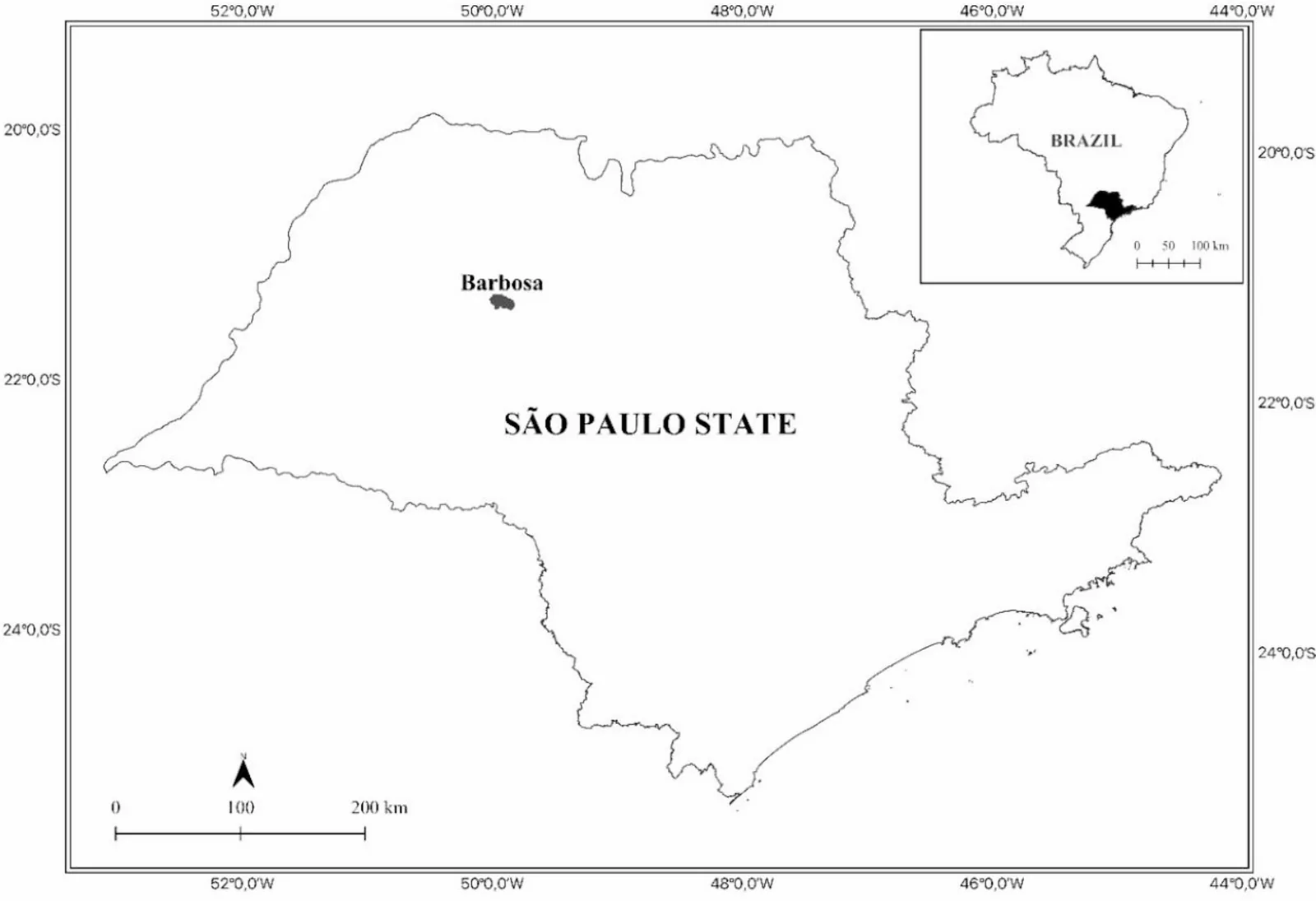
Study area in the Barbosa city (21°16′00″S, 49°56′57″W), São Paulo State, Brazil. The figure shows the geographic location of Barbosa within the state of São Paulo, in southeastern Brazil

The outbreak occurred in 2015 among workers at a slaughterhouse in Barbosa, a small town located in the state of São Paulo (Fig. 1).

The slaughterhouse primarily processed cattle, with occasional slaughter of water buffaloes. The facility employed 40 workers, including slaughter technicians and cleaning staff. Because the unit was small, all employees rotated through their duties and were involved both in cleaning the facilities and in handling slaughter materials.

### Clinical data collection

Blood samples for serologic testing were collected at the first visit, conducted in January 2015. There was no standardized treatment among patients: some received antibiotics, mainly rifampin or doxycycline, whereas others were not treated. The duration and dosage of antibiotic therapy varied. A second blood collection was performed 14 days later for titer comparison.

Clinical information was obtained from primary healthcare medical records provided by the Municipal Health Department of Barbosa and included symptoms at presentation, fever duration and intensity, hospitalization status, length of hospital stay, clinical complications, and antimicrobial therapy administered during the acute phase.

### Laboratory diagnosis

Before testing for Q fever, patients were screened for other infectious diseases, including dengue, brucellosis, leptospirosis, histoplasmosis, *Mycoplasma*, *Chlamydia*, and other pneumonia-causing agents, using the diagnostic tests routinely performed in the clinical investigation, and all results were negative.

Subsequently, serum samples were tested by indirect immunofluorescence assay (IFA) for detection of IgG antibodies using commercial kits (SCIMEDX Corporation, Denville, New Jersey, USA) containing phase II and phase I antigens of *C. burnetii* Nine Mile strain [22]. If serum was reactive at the 1:64 screening dilution, it was titrated at two-fold increments to determine endpoint titers to *C. burnetii* phases II and I.

Serum samples from all patients and sputum from the patients with respiratory symptoms were submitted to DNA extraction with QIAamp DNA Mini Kit (QIAGEN, Hilden, Germany) and tested by qPCR using a protocol adapted from Schneeberger et al. [23].

### Decade-later investigation

In 2025, a follow-up investigation was conducted to describe the long-term clinical and serological outcomes of the affected workers. Of the affected workers, 14/17 were located and agreed to participate in the study. The remaining three individuals had moved away from the region or could not be reached for contact.

The new serologic testing followed the same procedures and techniques used in 2015, and PCR was performed on whole blood samples instead of serum, following the protocol described by Schneeberger et al. [23] and Karounos et al. [24].

According to the CDC manual for the diagnosis of chronic Q fever, patients with clinical criteria and Phase I endpoint titers higher than 800, positive blood culture, or positive blood PCR are considered to have active and persistent infection and should be evaluated for endocarditis and other complications [2].

### Epidemiological analysis

A structured questionnaire was administered to healthcare professionals involved in patient care during the outbreak and to professionals currently working in the same health units in order to assess knowledge about Q fever, as shown in Supplementary Material (S1). Demographic variables of workers, including age, sex, and length of employment, were compared to evaluate potential differences between groups. Statistical analyses were conducted using SAS software (SAS, Cary, NC, USA), and a p value < 0.05 was considered statistically significant.

## Results

### Outbreak description (2015)

Of the 40 workers, 17 became ill in the same week, presenting high fever, abdominal pain, severe headache, and back pain, and sought medical care at local health units. The attack rate among workers was 42.5%, indicating that nearly half of the employees became ill during the outbreak.

Six patients required hospitalization due to the severity of symptoms. Fever was a common sign among all patients, with temperatures ranging from 40 °C to 42 °C. Supplementary Material (S2) presents the clinical symptoms of each patient, duration of hospitalization, acute disease complications, and treatment details. Length of stay in hospital ranged from 4 to 30 days.

Fever was high and recurrent, lasting from 10 to 40 days. Myalgia, headache, and abdominal pain were the most frequent symptoms, a typical pattern of acute Q fever, with variable respiratory manifestations and occasional gastrointestinal symptoms. Acute complications included pneumonia, hepatitis and urinary symptoms.

### Laboratory findings (2015)

Of 17 patients tested, 16 were seropositive in both the first and second collections. A four-fold or greater rise in endpoint titers to phase II antigens was observed in 14 patients (Table 1). Endpoint titers against *C. burnetii* showed higher phase II than phase I antibodies, a pattern consistent with acute infection.

**Table 1:** Serological results of 17 patients for IgG antibodies to *Coxiella burnetii* phase II and phase I antigens, tested in paired serum samples in 2015 (during an outbreak of Q fever) and as a single serum sample collected in 2025, in Barbosa, state of São Paulo, Brazil

Patients	2015				2025	
	1st sample		2nd sample		Single sample	
	Phase II	Phase I	Phase II^#^	Phase I	Phase II	Phase I
W1	128	128	128	128	-	128
W2	128	128	**1024**	128	-	64
W3*	512	128	**8192**	128	128	128
W4	2048	128	**8192**	4096	256	256
W5	128	128	**512**	128	-	1024
W6*	128	128	**512**	128	64	128
W7	128	-	**1024**	-	-	-
W8	512	128	**4096**	128	-	128
W9	2048	128	4096	128	-	256
W10	1024	128	**4096**	128	-	-
W11	512	128	**2048**	1024	128	512
W12	2048	2048	4096	4096	64	128
W13	-	-	**2048**	128	-	128
W14	128	128	**512**	512	256	512
W15	1024	128	**4096**	128	n.a	n.a
W16	2048	128	**8192**	128	n.a	n.a
W17	128	128	**2048**	128	n.a	n.a

-: sample non-reagent at the 1:64 serum dilution

n.a.: not available (patient was not found in 2025)

# titer values in bold indicate ≥four-fold rise in endpoint titers to phase II antigens between the 1st and 2nd serum samples in 2015

*PCR of patient sputum yielded *C. burnetii* DNA during the acute phase in 2015

All serum samples tested by qPCR were negative, whereas *C. burnetii* DNA was detected in sputum from two patients. Because some serum samples were collected within 5 days of symptom onset, positivity might have been expected; however, no standardized method exists for serum detection, which may not be the most suitable specimen. In the two patients with positive sputum, chest radiography showed bilateral pulmonary infiltrates.

### Decade-later investigation results (2025)

Overall, 12 of the 14 patients showed Phase I IgG endpoint titers for *C. burnetii* ranging from 64 to 1024; the later titer in a single patient. In the PCR analysis, all patients tested negative (Table 1).

Of the patients, 10/17 reported subsequent health problems after the outbreak (Supplementary Material), some of which are complications considered potentially associated with Q fever, including symptoms described in QFS [25, 26]. Of these, 8/10 reported extreme and abnormal fatigue shortly after the illness, which persisted to 2025. it was reported that common daily activities were impacted with inability to perform them in the same way.

Clinical data showed chronic alterations potentially associated with Q fever, such as dyspnea (28.6%, 4/14), cardiac disease (28.6%, 4/14), including endocarditis and pericarditis, and osteoarthritis (7.1%, 1/14). Other clinical alterations were reported, although their relationship with Q fever remains uncertain, such as systemic arterial hypertension (35.7%, 5/14), anemia (14.3%, 2/14), migraine (7.1%, 1/14), varicose veins (7.1%, 1/14), and premature birth (7.1%, 1/14).

Patients who did not receive specific treatment were advised to seek medical attention and clinical evaluation about a possible need of a new treatment regimen. Patients who developed significant complications in recent years, with or without serological evidence of chronic Q fever, were referred for clinical, laboratory, and echocardiographic re-evaluation.

### Epidemiological results

The affected workers included 12 men and five women, with a mean age of 30.2 years and a median employment duration of 3 years (range, 1–10 years) before the outbreak. No significant differences were observed between affected and non-affected workers regarding age, sex, or employment duration (*p* > 0.05).

According to employee’s reports, the animals slaughtered at the facility during that period included pregnant cows representing the main suspected source of the outbreak. Eighteen healthcare professionals were interviewed, including three who had been working in the local health units during the 2015 outbreak. All professionals interviewed reported that, at the time of the outbreak, they were unfamiliar with Q fever, including its clinical manifestations, transmission routes, and recommended treatment. Only professionals who experienced the outbreak reported seeking information about the disease and demonstrated awareness of its zoonotic nature.

## Discussion

Two female patients from the outbreak evolved to infective endocarditis and required cardiac surgery during long-term follow-up. Both had a family history of cardiovascular disease and were first-degree cousins.

In 2015, both had experienced pneumonia during the acute phase, and one had interrupted the proposed antimicrobial treatment. In 2025, one of them presented a phase I IgG titer of 1024, suggestive of chronic Q fever, while the other showed a phase I titer of 512. Information regarding laboratory results and antimicrobial treatment was not obtained when endocarditis was diagnosed. Cardiac complications are commonly associated with chronic Q fever, particularly endocarditis [25].

In 2025, four patients reported dyspnea and fatigue while performing simple activities. These symptoms may indicate a condition consistent with atypical pneumonia and should be investigated regardless of serological status [26].

In addition, only one woman from the cohort became pregnant after the outbreak. She had experienced prolonged acute symptoms in 2015 and, two years later, delivered a premature infant with Down syndrome and congenital heart disease. In 2025, she presented a phase I titer of 128, indicating residual antibodies.

The uniformity of responses in the structured questionnaire highlights a gap in knowledge and reinforces the importance of professional education to reduce diagnostic delays and mitigate the risk of future outbreaks. The lack of knowledge about Q fever among healthcare professionals observed in the present study reinforces one of the main challenges to the recognition of this neglected zoonosis. Limited awareness of its clinical manifestations, transmission routes, and appropriate diagnostic approaches may contribute to underdiagnosis, delayed treatment, and the progression to more severe clinical outcomes. Similar findings have recently been reported by Meurer et al. [27]. These findings emphasize the need for greater dissemination of information about Q fever, as well as the inclusion of this zoonosis in continuing medical education.

Cows usually shed infectious forms of *C. burnetii* during calving or abortion events, which has been the cause of several Q fever outbreaks reported in other countries [4, 5, 9]. The slaughter of pregnant cows represents a major risk for slaughterhouse workers, particularly for airborne diseases. A single bacterium can cause human infection; therefore, the inhalation of a large bacterial load likely had a much greater impact [2].

Therefore, the infection of the workers in 2015 likely occurred through inhalation of a larger inoculum and/or prolonged exposure to *C. burnetii* during the slaughter of pregnant cows, which may explain the severe symptoms and hospitalizations observed.

The occurrence of prolonged or recurrent fever lasting more than two to three weeks is a characteristic clinical feature of Q fever, as previously demonstrated [22]. Attention should be given to circulatory and cardiac complications in the chronic phase, as well as pneumonia and hepatitis in the acute phase. Patients with circulatory and cardiac problems in this study reported a family predisposition, suggesting a possible interaction between genetic susceptibility and disease progression, as previously evidenced in the study by Siciliano et al. [28].

It is possible that other workers at the site initially presented with asymptomatic or oligosymptomatic infections that were not recognized and subsequently developed symptoms associated with eventual persistent infection.

The recommended treatment for acute Q fever is doxycycline for 14 days, with follow-up to assess the need for extended or combined therapy. Rifampin, used during this outbreak, is not a recommended drug for the treatment of Q fever.

In some cases, doxycycline combined with another antibiotic is advised, but most patients did not receive the proper regimen [2], which may explain the occurrence of late complications such as endocarditis and the persistence of symptoms consistent with chronic Q fever.

This outbreak does not appear to be an isolated event, as a veterinary investigation on livestock farms in the same region of the outbreak detected infection in cattle milk and in ticks collected from cattle [29]. Additionally, in another region of the State of São Paulo, a 2022 study with febrile patients negative for dengue identified 21% cases of acute Q fever [22]. Furthermore, studies conducted in Southeastern Brazil, including Minas Gerais state, have reported acute Q fever in humans and infections associated with the consumption of contaminated cheese [30, 31].

The only follow-up study conducted years after Q fever infection was carried out in Germany, and it did not identify signs of chronic Q fever [32]. Our study suggests that the degree of exposure could be a potentially determining factor in explaining the occurrence of severe manifestations, since, unlike the German patients, those in this study worked directly with slaughter animals and were exposed to a potential high bacterial load. The absence of treatment or incorrect treatment are also determining factors.

It is important to acknowledge that some aspects should be considered when interpreting the results. First, only symptomatic workers were followed, as it was not possible to perform follow-up of asymptomatic individuals exposed during the outbreak. Therefore, it was not possible to determine the frequency of subclinical infections, persistent forms, or potential late complications among all exposed workers. In addition, the absence of a control group precluded comparisons of the frequency of the observed clinical manifestations, limiting inferences. Finally, the lack of systematic clinical and laboratory follow-up throughout the ten-year period between the outbreak and the patient reassessment limited the documentation of individual clinical evolution and the temporal confirmation of some reported complications.

Thus, future studies should include the longitudinal follow-up of exposed individuals, including asymptomatic workers, to better understand the frequency of persistent infections and long-term sequelae associated with Q fever. In addition, similar investigations involving patients evaluated years after outbreaks in different regions and countries, encompassing diverse epidemiological settings and occupational groups, may expand our understanding of the long-term clinical course of the disease. Finally, studies focusing on patients with endocarditis and pneumonia of unknown etiology may contribute to increasing the recognition of *C. burnetii* infection and reducing the underdiagnosis of Q fever.

Prevention measures and protocols for response during Q fever outbreaks is essential in slaughterhouses and animal farms worldwide, mainly because delayed diagnosis can impact patient treatment and recovery [33]. Due to the poorly understood epidemiology of the disease, the possibility of non-occupational or unconventional outbreaks should also be considered, as has already been described in prison, paper industry, pet transportation company, urban areas, and caves [6, 7, 10–12].

This is the first reported Q fever outbreak in Brazil and should be recognized by state and federal health authorities as a possibly non-isolated event, an occupational disease, and public health problem to be considered for rural and slaughterhouse workers in the country.

## Supplementary Information

Below is the link to the electronic supplementary material.

Supplementary Material 1

Supplementary Material 2

## Data Availability

The datasets generated and/or analyzed during the current study are available from the corresponding author on reasonable request.
