# Supplementary material for: Q fever outbreak in a slaughterhouse in Brazil: description and decade-later investigation
Source: Infection. 2026 Aug 24;54(5):2771–7. doi: 10.1007/s15010-026-02922-8 (PMC13727844; doi:10.1007/s15010-026-02922-8)
Supplement: Supplementary file 1 [file 15010_2026_2922_MOESM1_ESM.pdf]

Questionnaire for Healthcare Professionals — Q Fever Study

Full name: ______________________________ Date: ___ / ___ / ___

Healthcare unit: _____________________________

Occupation:
( ) Community health worker
( ) Nursing technician
( ) Nurse
( ) Doctor
( ) Other: ______________

Section 1 — Sociodemographic Data

1. Age: ______ years
2. Sex: ( ) Male ( ) Female ( ) Other: ______
3. Years working in public health: ______ years
4. Do you currently work in: ( ) Urban ( ) Rural ( ) Both

Section 2 — Involvement in the 2015 Outbreak

5. Were you working in healthcare in Barbosa in 2015?
   ( ) Yes ( ) No
6. Did you participate in any way in the investigation or care of patients during the outbreak?
   ( ) Yes ( ) No
7. If yes, how? (Check all that apply)
   ( ) Clinical care ( ) Sample collection ( ) Disease notification ( ) Home visits
   ( ) Technical meetings ( ) Others: ____________________________________

Section 3 — Knowledge about Q Fever

8. Had you heard about Q fever before this study?
   ( ) Yes ( ) No
9. How did you first learn about the disease?
10. ( ) Course ( ) News ( ) Experience during the outbreak ( ) Other: __________
11. What causes Q fever? What do you know about it? _________________________
12. Which routes of transmission are you aware of?
    ( ) Airborne transmission (contaminated dust)
    ( ) Consumption of raw milk
    ( ) Direct contact with animals
    ( ) Tick bite
    ( ) Person-to-person transmission
    ( ) I don't know

Section 4 — Clinical Practice and Training

13. Have you ever received specific training on Q fever?
    ( ) Yes ( ) No
14. If yes, when and where? ________________________________
15. In your opinion, Q fever is:
    ( ) A rare disease ( ) Endemic in some regions ( ) Relevant to public health
    ( ) Of secondary public health relevance
16. Do you know how to request laboratory testing for Q fever through the Brazilian Unified Health System (SUS)?
    ( ) Yes ( ) No
17. Have you cared for patients with suspected or confirmed Q fever after the outbreak?
    ( ) Yes ( ) No ( ) I don't know
18. When evaluating patients with compatible symptoms (prolonged fever, headache, atypical pneumonia, hepatitis, etc.), do you usually consider Q fever as a differential diagnosis?
    ( ) Yes ( ) No ( ) have never managed such cases

Section 5 — Perceptions and Occupational Health

19. Do you think Q fever poses a risk to healthcare professionals?
    ( ) Yes ( ) No ( ) I don't know
20. Have you experienced symptoms compatible with Q fever since 2015?
    ( ) Yes ( ) No
21. If yes, did you undergo laboratory testing?
    ( ) Yes ( ) No ( ) I don't remember
22. Do you know any healthcare professionals who became ill during the outbreak?
    ( ) Yes ( ) No
23. In your opinion, did the 2015 outbreak have a significant impact on the health of the local population?
    ( ) Yes ( ) No ( ) I don't know
24. Would you like to receive more information or training about Q fever and other neglected zoonotic diseases?
    ( ) Yes ( ) No

Section 6 — Perceptions and Attitudes of Colleagues

25. During the 2015 outbreak, did you hear healthcare professionals questioning the diagnosis of Q fever?
    ( ) Yes ( ) No ( ) I don't remember
26. In your opinion, healthcare professionals at that time:
    ( ) Took the outbreak seriously
    ( ) Considered it an exaggeration or mass hysteria
    ( ) Demonstrated lack of knowledge and disbelief regarding the disease
    ( ) I don't know / Prefer not to answer

27. In your opinion, was there any negligence or lack of adequate care provided to the affected workers?
( ) Yes ( ) No ( ) I don't know
28. Have you ever witnessed situations in which patients diagnosed with Q fever were discredited, ridiculed, or poorly treated by healthcare professionals?
( ) Yes ( ) No ( ) I am not aware of any such situations
29. How do you think the management of Q fever could have been better conducted by the healthcare services at that time? Please include any additional comments or experiences.
____
____
____

__________________________ (Participant)

__________________________ (Interviewer)

Prof. Dr. Danilo França. Veterinarian and Researcher.
